# Artificial Intelligence in Gastroenterology—Walking into the Room of Little Miracles

**DOI:** 10.3390/jcm9113675

**Published:** 2020-11-16

**Authors:** Wojciech Marlicz, George Koulaouzidis, Anastasios Koulaouzidis

**Affiliations:** 1 Department of Gastroenterology, Pomeranian Medical University, 71-252 Szczecin, Poland; 2 The Centre for Digestive Diseases Endoklinika, 70-535 Szczecin, Poland; koulaou@yahoo.co.uk; 3 Endoscopy Unit, The Royal Infirmary of Edinburgh, Edinburgh EH16 4SA, UK; akoulaouzidis@hotmail.com

The surge of artificial intelligence (AI) in medicine stands on a lengthy, and frequently reticent, buildout. AI is an all-encompassing term for techniques that enable the software to mimic human intelligence (HI) using logic, convolutional neural networks (CNN), decision trees, search algorithms, machine learning (ML) and deep learning (DL) [1]. ML creates mathematical learning algorithms (MLAs) from given datasets and utilizes them to predict various outcomes and/or facilitate decisions. However, MLAs and DL have only recently drawn the spotlight, attracting the attention of gastroenterologists and hepatologists. Recently, AI solutions have been explored to better define mucosal healing in inflammatory bowel disorders (IBD) [2]. With the incidence and global burden of IBD still on the rise, there is a constant need for more accurate ways to stratify risks and predict prognosis in these patients. 

Therefore, of special interest is the paper by Choi et al., who aimed to develop and validate MLAs to predict the 5-year risk of starting biologic agents in IBD patients, published in this Special Issue of Journal of Clinical Medicine (JCM) “AI in Medical Practice/Gastroenterology, Hepatology and Endoscopy” [3]. To attain their aim, the authors applied an ML method to the database of the Korean common data model (K-CDM) network, a data-sharing consortium of tertiary medical centers in South Korea. ML as a methodology offers several advantages over traditional statistical approaches to explore large datasets to develop prediction models of cancers or pre-cancerous gastrointestinal (GI) lesions. However, to date, no ML algorithm has been developed for clinical studies looking at IBD outcomes, especially in Asian countries. Choi et al. utilized an ML model encompassing a support vector machine (SVM) (non-linear model), random forest (RF), XGBoost (XGB), artificial neural networks (ANN), as well as ensemble methods for the prediction of an IBD-related outcome (commencement of biologic agents within 5 years of diagnosis) using clinical and laboratory data from GMC and the K-CDM network in Korea [3]. This study is important as it represents the first attempt to apply an ML algorithm to predict outcomes in IBD patients, which could grant physicians valuable insight into the characteristics of high-risk patients. MLAs also allow early clinical intervention for IBD patients, with the intending to minimize disease-related adverse events. 

The appearance of automated lesion recognition endoscopy software combined with the boost in robotic techniques in surgery [4] has captured the attention of GI endoscopists and surgeons. For example, clinical risk-scoring systems offer invaluable, but not always practical, help for better stratification of patients at risk of upper GI bleeding (UGIB) and hemodynamic instability. As reported in this Special Issue, Seo et al., developed an ML algorithm that predicts adverse events in patients with initially stable non-variceal UGIB [5]. The authors’ primary outcomes included adverse events such as mortality, low blood pressure and rebleeding within 7 days. The authors compared four MLAs: logistic regression with regularization (LR), random forest classifier (RF), gradient boosting (GB) classifier, and voting classifier (VC) with clinical Glasgow–Blatchford (GBS) and Rockall scores. They found the RF model was of the highest accuracy and offered significant improvement over conventional methods for predicting mortality and VC for hypotension and rebleeding within 7 days. Their data call for the implementation of MLAs into electronic health records (EHR) as an early predictive tool for the stratification of initially stable high-risk UGIB patients before, or at the time of, admission to the endoscopy department [5]. 

AI has already supported wireless capsule endoscopy (CE) platforms in the diagnosis of small bowel (SB) bleeding, as well as medical practitioners in the assessment of patients with liver fibrosis or chronic pancreatitis suspected of cancerous progression. AI has been continuously explored for the last decade to facilitate the detection of pre-cancerous and cancerous mucosal GI lesions. In GI endoscopy, AI has become an even hotter topic in recent years, as the majority of manufacturers started to update their systems to accommodate the new needs. Undoubtedly, AI has come to ’democratize’ GI endoscopy by improving patients’ outcomes irrespective of place/time of care, enhancing the professional experience for clinicians, and assisting healthcare systems with capacity issues. Yang et al. [6] significantly contributed to this field, demonstrating the development and validation of DL models that automatically classify colorectal lesions histologically on white-light (WL) colonoscopy images. The authors in the paper presented in the current JCM Special Issue collected WL colonoscopy images of colorectal lesions exhibiting pathological results and classified them into seven categories: (i) stages T1–4 colorectal cancer (CRC), (ii) high-grade dysplasia (HGD), (iii) tubular adenoma (TA), (iv) and non-neoplasms. Next, they re-classified the images into four categories including (i) advanced CRC, (ii) early CRC/HGD, (iii) TA, and (iv) non-neoplasms. They trained two CNN followed by the evaluation of performances in an internal test dataset and an external validation dataset [6]. The authors for the first time established a CNN model with a promising performance in classifying colorectal neoplasms from non-neoplastic lesions to advanced CRC based on standard WL colonoscopy images. Their CNN model could be adopted to assist in the accurate prediction of histology and decision-making to further manage colorectal lesions in the real-life endoscopy unit [6].

AI has been instrumental in big data analyses in the era of -omics technologies, targeting microbiomes, metabolomes and other complex living ecosystems. In an era where information overload has become the norm [7], the number of publications is skyrocketing and science moves with exponential pace, equal outcomes remain a challenge. Not every institution is endowed with experts in all fields, nor is it possible to understand novel data to implement them into daily medical practice. Therefore, of interest is the scoping review of Goyal et al., addressing AI in the screening and diagnosis of colorectal cancer, published in the current JCM Special Issue [8]. The authors review and discuss various AI and CAD applications in colorectal cancer screening, polyp detection, and characterization in various settings and topics, such as (i) CT colonography (CTC), (ii) colon capsule endoscopy, (iii) magnifying narrow-band imaging (NBI), (iv) magnifying chromoendoscopy, (v) endocytoscopy, (vi) confocal endomicroscopy/confocal laser endomicroscopy, (vii) laser-induced fluorescence spectroscopy (LIFS), and (viii) autofluorescence endoscopy [8].

Following the outbreak of the novel COVID-19 disease, there has been a drastic reduction in elective healthcare services, including diagnostic endoscopy services, as these carry the risk of communicable viral disease transmission. These reductions for all but the most urgent clinical work have a detrimental impact not only on patients but also on practicing physicians. As the diagnostic and therapeutic capabilities of GI endoscopy strongly correlate with the skills of the operator, the altered learning curves required to achieve proficiency in endoscopic techniques play roles. Moreover, increasing numbers of physicians/nurses present burnout syndromes. Furthermore, increasing numbers of medical staff in GI units report endoscopy-related muco-skeletal injuries. Last but not least, at a time of the pandemic, there is a constant shortage of human and material resources. These all lead to the worsening of patients’ and physicians’ perspectives on contemporary GI endoscopy practice. Those factors seriously interfere with the successful implementation, adoption and continuation of daily medical routines. They also have a negative effect on the number of diagnostic screening and therapeutic procedures. Therefore, various AI solutions to enhance lesion detection and short-/long-term outcome prediction must be rapidly implemented in daily GI practice. It will benefit both physicians and their patients, as well as supporting the overstrained healthcare systems. Of further relevance are the recent advancements in other medical technologies. With patients’ growing expectations for high-tech precision diagnostics, GI endoscopy has also been successfully utilized for the innovative detection/treatment of GI pathologies. For this purpose, robotic gastroscopes and colonoscopes with advanced functionalities have been developed and mastered [4,9]. The implementation of AI technology in these novel robotic devices, combined with remote telehealth services, will further support the medical practitioner and healthcare units. In parallel, there is also a great need for new AI educational software, mobile applications, and virtual reality (VR) tools, which would allow the managing of medical complexities, as well as helping in coping with the stress and fatigue that is rife among healthcare professionals [9,10]. It is certain that AI already aids in building trust and credibility among patients, as it allows for better, faster and more accurate diagnosis and treatment outcomes. 
